# Patients With Implantable Cardioverter Defibrillators on Social Media Report More Shock Anxiety Than Clinic Patients: Results From an Online Survey

**DOI:** 10.2196/cardio.8152

**Published:** 2017-09-26

**Authors:** Linda Kramer Freeman, Keith Richards, Jamie B Conti, Samuel F Sears

**Affiliations:** ^1^ Carlson School of Management University of Minnesota Minneapolis, MN United States; ^2^ School of Communication East Carolina University Greenville, NC United States; ^3^ Division of Cardiovascular Medicine University of Florida Gainesville, FL United States; ^4^ Departments of Psychology and Cardiovascular Sciences East Carolina University Greenville, NC United States

**Keywords:** health communication, social media, implantable cardioverter-defibrillator, cardiology patients, shock anxiety

## Abstract

**Background:**

Coping with heart disease and the potential for implantable cardioverter defibrillator (ICD) shocks challenges the psychological adjustment of patients with ICDs. Social media use may be used to seek education and support from others.

**Objective:**

The aim of this study was to examine the content of information sought online and whether a social media sample of patients with ICDs report more device-specific anxiety than clinic-based normative samples.

**Methods:**

A total of 196 participants were recruited via social media messages and invited to complete an online survey.

**Results:**

It was found that the information most often sought by online users (62.4%, 123/196) involved both emotional support (eg, gaining emotional support from other patients with ICDs) and technical information (52.6%, 103/196) (eg, dealing with magnetic interference). The online sample reported more shock anxiety than a typical clinical sample with mean values of 22.75 (SD 10.06) and 15.18 (SD 6.50), respectively (*P*<.001).

**Conclusions:**

Collectively, these results suggest that patients with ICDs that are online are seeking emotional information and support, and that they report increased shock anxiety relative to typical clinic-based patients. Future research should examine how online information and clinical-based information form a composite understanding and adjustment for patients ICDs.

## Introduction

### Background

The implantable cardioverter defibrillator (ICD) has demonstrated successful reduction of mortality in patients at risk for life threatening arrhythmias. Living with an ICD includes managing the stress of cardiac disease, accepting the ICD, and minimizing distress [1]. The unique aspect of living with an ICD is the possibility of experiencing a painful high-energy shock necessary to terminate a potentially life-threatening cardiac arrhythmia. Psychological distress is common, with approximately 20% of patients reporting anxiety and/or depression [2].

Risk and resilience factors for psychological distress have also been established. Individuals who are younger than age 50, women, and having experienced an ICD shock are known risk factors. Resilience factors include optimism, faith in their ICD, and confidence in their doctor [3]. Patients with ICDs may attempt to address their concerns via social media. Non-randomized, Internet-recruited patients can provide insight and potentially help ICD providers to clarify issues in clinic that are currently primarily addressed by social media.

### Social Media as a Health Communication Resource

Patients are increasingly using social media to share information and support with other patients and experts. These interactions may help individuals learn about what they should be expecting as well as ways to cope with their health issues [4]. Health issues explored include social support and advice [5], mental health concerns [4], diabetes [6], and public health [7]. Nearly 70% of Americans use social media and 68% of adults use Facebook [8]. A meta-analysis revealed the benefits of health communication via social media include increased interaction, shared and tailored information, increased access to health information, and peer, social, and emotional support [9]. Understanding how communicating about ICD via social media benefits patients with ICDs could prove valuable for disseminating information and meeting the needs of this unique population.

### Objectives

The objectives of this study were (1) to examine what information is sought via social media by patients with ICDs; and (2) to determine whether a social media sample of patients with ICDs report more device-specific anxiety than clinic-based normative samples. This information is important for creating a greater understanding of what information is valuable to patients with ICDs and better understanding how anxiety influences their information seeking. Together, these could lead to improvements in the quality of life of all patients with ICDs by providing the information they value most, which may decrease their anxiety.

## Methods

### Participants

The non-randomized sample consisted of 196 patients with ICDs recruited through Facebook groups that were affiliated with heart disease and ICD topics. Facebook was chosen over other social media sites because patients with ICDs on this site engaged with one another on a more consistent basis and were more receptive to the survey. Researchers created a Facebook profile that identified the principal investigator and created posts with the details of the study including the following requirements for participation: individuals who currently had an ICD and who were at least 18 years of age. Participants were informed that if they completed the survey they would be eligible for a US $25 Amazon gift card. The recruitment period was June 2014 through January 2015. Participants were asked to complete standardized and validated questionnaires in an online survey. The study, funded by a university grant and approved by the Institutional Review Board (IRB), was made electronically available to participants after obtaining informed consent.

Two strategies prevented those without an ICD from participating in the survey. First, individuals were asked if they had an ICD. Those who answered “no” were immediately removed from the survey. Second, individuals were asked to indicate the brand of their ICD. If they did not know, they too were immediately removed.

### Demographic Variables

Information on gender, age, and educational level was obtained from the survey. The amount of time since the implant of the ICD, how recently the patient had experienced ICD-administered shock, and the number of ICD shocks were also assessed in the survey.

### Shock Anxiety

The Florida Shock Anxiety Scale (FSAS) contains 10 items that contribute to the subscales “triggers” and “consequences”. A total score is determined by summing the items. The FSAS is a reliable measure (alpha = .925). Respondents rate each item on a 5-point Likert scale ranging from 1 (not at all) to 5 (all the time). Higher scores indicate greater shock anxiety. The FSAS has been utilized as a shock anxiety measure internationally with studies in Australia [10] and Canada [11].

### Social Media Use

Participants were asked to identify who they discussed the ICD with through social media. Participants selected from a list that included doctors, nurses and other healthcare providers, patients, friends, and “other.” A list of common concerns of patients with ICDs was provided along with the prompt:

What type of information or support about ICD have you looked for online or discussed on social media? Select all the reasons that apply

Participants were also asked which sites they visited to discuss ICDs (ie, Facebook, Twitter, Pacemaker Club, YouTube, American Heart Association, and “other”).

### Recent Shock

Participants were asked to indicate when they had received their last shock and the following groups were created: (1) Group 1, those whose most recent shock occurred less than 4 months ago; (2) Group 2, those whose most recent shock occurred more than 4 months ago; and (3) Group 3, those who had never experienced a shock from their ICD.

### Statistical Methods

Categorical variables were compared between groups using the Pearson chi-square or Fisher test with frequencies and percentages reported. Continuous variables were compared between groups using the Student *t* test. All tests were 2-tailed with significance level of .05. Analyses were performed with SPSS version 20. When the Levene test for the assumption of homogeneity of variance was not met for this data, Welch statistics were used for calculating and reporting degrees of freedom.

## Results

### Demographics

The sample consisted of 196 patients with an ICD that completed the survey: 130 (66.3%, 130/196) were women and 66 (33.7%, 66/196) men. Participant age ranged from 18 to 70 with an average age of 45.61 (SD 12.54); 60.1% (113/188) were under age 50. The amount of time since implant of the ICD were as follows: 18.4% (36/196) had the ICD for less than 1 year, 25.0% (49/196) for 1 to 2 years, 27.6% (54/196) for 3 to 5 years, 14.8% (29/196) for 6 to 10 years, and 14.3% (28/196) for more than 10 years. The sample was almost entirely White (91.3%, 179/196) with 85.2% (167/196) reporting at least some post-secondary education.

### Implantable Cardioverter Defibrillator Concerns

A list of topics related to the ICD and the percentage of survey participants who indicated that they go online or use social media to discuss the topic is shown in Table 1.

Participants were asked to respond to the following and were supplied a list of 20 possible topics:

What type of information or support about ICD have you looked for online or discussed on social media? Select all the reasons that apply.

The most common topics selected were social support and shock anxiety, with more than half of the sample, 62.8% (123/196) and 55.6% (109/196) selecting the items “gaining emotional support from others going through the same thing as me” and “anxiety about my ICD”, respectively, as reasons why they chose to use social media.

The gender distribution of incidence of shock is shown in Table 2. Almost half of the participants (49.0%, 96/196) had experienced at least 1 shock from their ICD, with 40.8% (53/130) of women and 65% (43/66) of men indicating that they had been shocked at least once.

### Shock Anxiety Comparisons

Comparisons on shock anxiety between the online sample and the typical clinical sample [12] confirmed that the online ICD group reported higher shock anxiety (*t*_189_ = 10.36, *P*<.001) (Table 3). Analysis of variance (ANOVA) revealed that recent shock was also a significant factor in shock anxiety. A main effect on shock anxiety for those experiencing the most recent shock was found (*F*_2,186_= 33.19, *P*<.001).

**Table 1:** Communicating about implantable cardioverter defibrillator topics through an online social media modality (N=196).

Topic	Participants, n (%)
Gaining emotional support from others going through the same thing as me	123 (62.8%)
Anxiety about my ICD^a^	109 (55.6%)
Information about magnetic interference	103 (52.6%)
Keeping up-to-date on ICD news	89 (45.1%)
Camaraderie with others	84 (42.9%)
Travel or vacation with ICD	82 (41.8%)
Fatigue	79 (40.3%)
Exertion while exercising	79 (40.3%)
Activity restrictions	74 (37.8%)
Fear of shocks	68 (34.7%)
Device recalls	68 (34.7%)
Expectations for the future	54 (27.6%)
Sports participation	46 (23.5%)
Appearance of ICD	46 (23.5%)
Pain	40 (20.4%)
Family matters	35 (17.9%)
Concerns about sexual activity with ICD	25 (12.8%)
Body image concerns	19 (9.7%)
ICD and pregnancy	17 (8.7%)
Other	15 (7.7%)

^a^ICD: implantable cardioverter defibrillator.

**Table 2:** Participant's most recent shock by gender (N=196).

Gender	Most recent shock, n (%)		
	Less than 4 months ago	More than 4 months ago	Never
Women (n=130)	12 (9.2%)	41 (31.5%)	77 (59.2%)
Men (n=66)	19 (28.8%)	24 (36.4%)	23 (34.8%)

**Table 3:** Summary of means, standard deviations, and standard errors for scores on the Florida Shock Anxiety Scale (FSAS).

Variable	n (%)	Mean (SD^a^)	SE^b^
FSAS^c^ total sample	190 (100%)	22.75 (10.06)	
FSAS clinic^d^	443	15.18 (6.50)	
FSAS male	61 (32.1%)		1.49
FSAS female	122 (64.2%)		0.78
FSAS age less than 50	109 (57.4%)		0.98
FSAS age greater than 50	74 (38.9%)		0.98

^a^SD: standard deviation.

^b^SE: standard error.

^c^FSAS: Florida Shock Anxiety Scale.

^d^Clinical results from Ford et al, 2012. All other FSAS scores reported reflect the current sample.

Shock history was further investigated related to 3 recent shock conditions: Group 1 (less than 4 months ago); Group 2 (more than 4 months ago); and Group 3 (never shocked). Bonferroni pairwise comparisons revealed significant differences between Group 1 compared to Group 2 with a mean difference of 13.07 (*P*<.001), and Group 1 compared to Group 3 with a mean difference of 14.79 (*P*<.001). The pairwise comparison between Group 2 and Group 3 was not significant.

## Discussion

### Principal Findings

This study revealed that the most common reasons to go online are to gain emotional support from others and to express anxiety to the community of patients with ICDs. Communicating about their ICD through social media allows patients to share their interests and concerns with others who are similarly interested in discussing the ICD, beyond existing face-to-face support groups and visits to a healthcare clinic. Benefits include offering and receiving practical advice, support, and meaningful information related to all aspects of living with the device [13]. Patients with ICDs can connect with others online immediately to anonymously obtain information about the ICD or to provide and/or receive social support.

### Online Health Information

Along with the benefits accrued from social media interaction about ICD, there may be some drawbacks. Group norms that develop in online ICD groups may be beneficial or harmful for participants and should be studied. Information shared online may be inaccurate or biased [9]. Misinformation can quickly spread through social media, with the potential to raise anxiety in patients with ICDs. Future research should explore the best ways to monitor the accuracy, validity, and reliability of the ICD information shared online through social media. Presumably the effects of misinformation could be mitigated by seeking multiple sources of information and through discussions with their provider [14]. Other areas of interest relate to time since implantation as patients with an ICD for longer than 1 year have had time to broaden their online engagement regarding their ICD. During the first year after implant of the device, patients have frequent meetings with healthcare providers to ensure the device is working properly and to assuage patient concerns. In subsequent years, patients are increasingly independent, relying on remote monitoring and less frequent visits with healthcare providers. It is reasonable to assume patients would increase their reliance on social media for support and information after the first year.

### Online Shock Anxiety

The current study indicated that patients with ICDs who use social media to communicate about their ICD reported significantly greater shock anxiety than the general population of patients with ICDs. Highly anxious patients may be more likely to overestimate the personal risk of adverse events from generic information or probabilities without information specific to their condition. Nonetheless, online engagement may be the most accessed information for these patients because it is always available. These results suggest that clinicians may want to increase their online information offerings to include both information and a “relevance test” of information for patients to contact clinicians about specific probable risks versus possible risks and anxieties about their ICD. Clinicians should also inquire further about what information patients preferentially used Internet sources to obtain instead of in-clinic conversations. Ideally, multiple, reliable sources could be accessed as needed to learn about technical issues and more personal issues such as emotional functioning or supportive conversations.

### Limitations

The current study has some limitations to consider while interpreting its results. Because the participants were recruited through Facebook groups, they had online information-seeking skills and experience communicating through the Internet, possibly influencing their choices regarding the sharing of ICD information and thoughts online. Despite efforts to recruit participants from a variety of social media sites such as YouTube, Twitter, and Instagram, only Facebook groups yielded individuals willing to complete the survey, which may limit the results to those who are active participants in social media groups and chose to participate in the survey. Future research should expand the number and variety of participants by recruiting from additional social media sites to assess whether the results are generalizable across the social media landscape. Whether the patients with ICDs who participated in the study are representative of the population of patients with ICDs who use social media to communicate about their device cannot be determined. The conclusions in this study show only associations, not causation. In addition, although researchers attempted to screen out individuals who did not have an ICD, it is possible that an individual could provide false answers to gain access to the proffered incentive. Answers to the survey were self-reported, relying on the integrity of the participants to respond to survey questions honestly and accurately. Further, the current study did not control for previous history of medical and/or psychological difficulties that may have impacted the results and reduced the general application of results.

### Conclusion

This study examined the content of information sought online and whether a social media sample of patients with ICDs report more device-specific anxiety than clinic-based normative samples. Patients with ICDs most often sought information focused on both emotional support (62.8%, 123/196) and technical information (52.6% 103/196). This study of patients with ICDs recruited online indicated higher levels of shock anxiety than a typical clinical sample. Higher shock anxiety was associated with recent shock. This study demonstrated that patients with ICDs seek up-to-date information and emotional support on social media, and younger patients are increasingly likely to use social media to discuss their ICD concerns. While patients with ICDs have access to face-to-face healthcare professionals in clinics and support groups, there is substantial interest among patients to share information and support through social media. Delivery of high quality, appropriate, cost effective online support for patients with ICDs offers the potential for better psychological adjustment to the realities of life with an ICD.

## Abbreviations

ICD: implantable cardioverter defibrillator
FSAS: Florida Shock Anxiety Scale
